# Knowledge, Attitudes, and Practices Regarding Breast Cancer Screening Among Females in Saudi Arabia

**DOI:** 10.3390/healthcare14132003

**Published:** 2026-07-06

**Authors:** Nawaf W. Alruwaili, Abdullah Mohammed Alfehaid, Khaled Abdullah Shafi Al-Toum, Aljazi Bin Zarah, Nora Alafif

**Affiliations:** 1 Department of Community Health Sciences, College of Applied Medical Sciences, King Saud University, Riyadh 11433, Saudi Arabia; 445911983@student.ksu.edu.sa (A.M.A.); kaltoum@moh.gov.sa (K.A.S.A.-T.); abinzaraah@ksu.edu.sa (A.B.Z.); nalafeef@ksu.edu.sa (N.A.); 2 Public Health Authority, Riyadh 13352, Saudi Arabia; 3 Ministry of Health, Riyadh 12822, Saudi Arabia

**Keywords:** breast cancer, mammography, screening, knowledge, attitudes and practices, Health Belief Model, mammography barriers, Saudi Arabia

## Abstract

**Background:** Breast cancer comprises 31.4% of all female cancers in Saudi Arabia (2020 Cancer Registry). Despite free national screening services existing since 2005, mammography utilization remains critically low. This study assessed breast cancer knowledge, attitudes, and practices (KAP) among females in Saudi Arabia and identified independent predictors of screening behavior. **Methods:** A cross-sectional study (December 2024–February 2025) enrolled 426 females aged ≥20 years from all 13 Saudi administrative regions using a quota-based design combining facility-based and online recruitment. Attitude and barrier domains were adapted from Champion’s Health Belief Model Scale (CHBMS), validated in Arabic; knowledge items used validated regional instruments. Knowledge-score reliability: KR-20 = 0.45; attitude subscale: α = 0.74. Binary logistic regression identified independent predictors of screening uptake (outcome: any screening in the preceding five years, coded as screened = 1; not screened = 0). **Results:** Mean composite knowledge score: 4.51 ± 1.52/7 (KR-20 = 0.45); 54.0% achieved high knowledge (≥5). Mammography uptake was 30.5% overall and 52.2% among women aged ≥40 (*n* = 136; the recommended target group). Predominant barriers: Fear of diagnosis (83.6%), belief in incurability (76.3%), radiation concern (73.2%), and pain anxiety (72.3%). Logistic regression (χ^2^(8) = 188.96, *p* < 0.001; McFadden’s pseudo R^2^ = 0.323) identified older age (OR = 1.52; 95% CI: 1.21–1.92), higher income (OR = 1.57; 95% CI: 1.25–1.99), transportation barriers (OR = 3.39; 95% CI: 1.95–5.89), and family discouragement (OR = 3.03; 95% CI: 1.72–5.34) as significant predictors (all *p* < 0.001). **Conclusions:** A significant knowledge–practice gap persists across all 13 Saudi regions. These findings suggest several implications for a multi-level public health response to be evaluated through future intervention research; multi-level strategies targeting CHBMS Barriers are needed.

## 1. Introduction

Breast cancer is the most prevalent malignancy among women worldwide. According to the most recent GLOBOCAN 2022 estimates, breast cancer accounted for 11.6% of all newly diagnosed cancers (both sexes combined) and 6.9% of all cancer deaths in 2022, with approximately 2.3 million new cases and an estimated 666,000–670,000 deaths recorded globally that year [1]. Five-year survival rates diverge substantially—exceeding 99% for localized (stage I) disease yet falling to approximately 29% for distant (metastatic) disease [2]—a disparity attributable primarily to late-stage presentation and inadequate screening coverage [3,4].

In Saudi Arabia, breast cancer has exhibited a sustained increase in incidence. The age-standardized incidence rate (ASR) rose from 12.6 to 49.7 per 100,000 women between 2002 and 2022—an average annual percent change (AAPC) of 5.6% (95% CI: 4.5–6.7) [5]. According to the Saudi Cancer Registry 2020 report, breast cancer accounted for 31.4% of all cancers recorded among women, with 2459 new female cases and an ASR of 28.4 per 100,000 [6]. Saudi women are typically diagnosed at a younger median age (~50–52 years) and present disproportionately with locally advanced or metastatic disease [7,8]. These figures surpass historical 2008–2014 estimates [9], confirming an accelerating incidence. The WHO Global Breast Cancer Initiative (WHO GBCI), launched in 2021, specifies three pillar-specific key performance indicators rather than a population mammography-coverage target: Pillar 1 (health promotion and early detection) targets ≥60% of invasive breast cancers diagnosed at stage I or II; Pillar 2 targets diagnostic completion within 60 days of presentation; and Pillar 3 targets ≥ 80% treatment completion without abandonment [10]. No national stage-distribution data are available to benchmark Saudi Arabia against Pillar 1 directly, but the low screening uptake reported below suggests this target is unlikely to be met without expanded early detection efforts.

The Saudi Ministry of Health (MOH) recommends monthly breast self-examination (BSE), annual clinical breast examination (CBE) from age 40, and biennial mammography for women aged 40–74 years [11]. Despite nationwide free screening since 2005, a 2015 national survey found that 92% of eligible Saudi women had never undergone mammography [12]. The socioeconomic dimension is substantial: mammography uptake is significantly more concentrated among wealthier and better-educated Saudi women (income concentration index = 0.163) [5], confirming that the free-service model alone does not eliminate structural inequities.

Knowledge, attitude, and practice (KAP) studies offer an established epidemiological framework for identifying the cognitive and behavioral determinants of preventive health uptake. The Health Belief Model (HBM)—operationalized through Champion’s Health Belief Model Scale (CHBMS) [13,14], and validated in Arabic by Mikhail and Petro-Nustas [15]—posits that health behavior is governed by perceived susceptibility, severity, barriers, benefits, and self-efficacy [16]. Prior Saudi and Arab-world KAP research has identified psychological barriers (fear of diagnosis, pain and radiation anxiety), sociocultural constraints (embarrassment, familial opposition), and structural obstacles (inaccessibility, transportation) as primary impediments to breast cancer screening [17,18,19]. A 2025 literature review found that poor knowledge, negative attitudes, and limited screening practices are endemic across the MENA region [20]. However, the preponderance of existing Saudi studies is confined to single cities or institutions, substantially limiting their generalizability across the country.

The present study addressed this evidence gap by examining breast cancer KAPs across all 13 administrative regions of Saudi Arabia. Specific objectives: (i) Assess composite breast cancer knowledge and awareness of screening modalities; (ii) evaluate attitudes toward breast cancer screening; (iii) document current screening practices, including age-stratified mammography uptake; and (iv) identify independent sociodemographic and attitudinal predictors of screening behavior.

## 2. Materials and Methods

### 2.1. Study Design and Setting

A cross-sectional study was conducted from December 2024 to February 2025 across all 13 administrative regions of Saudi Arabia: Riyadh, Makkah, Madinah, Eastern Province, Qassim, Tabuk, Najran, Ha’il, Northern Borders, Al-Jouf, Asir, Jazan, and Al-Baha. Data were collected at primary healthcare centers (PHCs), hospitals, and community centers, supplemented by an online platform distributed via the King Saud University (KSU) institutional email distribution list and social media platforms. This study adheres to the STROBE guidelines for cross-sectional studies (Supplementary File S2).

### 2.2. Study Population and Sampling

The target population comprised females aged ≥20 years residing in Saudi Arabia, including both Saudi nationals and resident non-Saudi women. A quota-based design was used: recruitment targets were pre-specified for each of the 13 administrative regions and for five age strata (20–29, 30–39, 40–49, 50–59, ≥60 years); within each region/age-stratum cell, participants were recruited through a combination of facility-based (PHC, hospital, and community center) and online (KSU institutional email and social media) channels until the target was reached. This approach achieved broad geographic and age-group coverage but does not constitute probability sampling from a complete national sampling frame; consequently, findings should not be interpreted as nationally representative in the strict statistical sense (see Section 4.5). The minimum sample size was calculated using Cochran’s formula: *n* = Z^2^pq/d^2^ (Z = 1.96, *p* = q = 0.50, d = 0.05), yielding *n* = 384, which was inflated by 10% to *n* = 426—achieved in full.

### 2.3. Data Collection Instrument and Validated Sources

A structured, self-administered Arabic questionnaire was developed through systematic adaptation from published, validated instruments (Supplementary File S1).

#### 2.3.1. Attitude Domain—Champion’s CHBMS

The six-item Likert attitude subscale (1 = Strongly Disagree to 5 = Strongly Agree) was adapted from Champion’s CHBMS Benefits and Barriers subscales [13,14], with Arabic validation by Mikhail and Petro-Nustas [15] in 519 Jordanian women. Internal consistency: Cronbach’s α = 0.74, consistent with Saudi CHBMS adaptations [21,22].

#### 2.3.2. Barrier Domain—Revised Barrier Scale

The ten-item barrier section was adapted from Champion’s Revised Barriers to Mammography Scale [14], validated in Arabic [15]. Four Saudi-specific items were appended: belief in incurability [23]; belief that screening is unnecessary without family history [7]; transportation difficulties [18]; and family discouragement [17].

#### 2.3.3. Knowledge and Awareness Domain

The 19-item knowledge section was adapted from Alshahrani et al. [7] (Najran PHC patients) and Heena et al. [21] (Saudi healthcare professionals; α = 0.71–0.79), cross-referenced with Alghamdi et al. [23]. Awareness items assessing symptom recognition were informed by the Breast Cancer Awareness Measure (BCAM) symptom recognition subscale and help-seeking intention items [24].

#### 2.3.4. Instrument Validation

Content validity: Expert panel of three specialists; mean item CVI = 0.91 (threshold ≥ 0.80). Pre-tested in 20 women; no structural revisions required. The seven items selected for the composite knowledge score (the remaining 12 of 19 knowledge-domain items assess awareness engagement, information sources, and mammography-specific knowledge) were the core factual/biomedical items suited to a summed correct-count index: disease awareness, treatability, contagion misconception, three modifiable risk factors, and early detection awareness. For this dichotomous (correct/incorrect) item set, the Kuder–Richardson 20 (KR-20) coefficient—mathematically equivalent to Cronbach’s α for dichotomous items—was recomputed directly from the raw response data and found to be 0.45. Item-total correlations (corrected r) ranged from −0.11 (the contagion-misconception item) to 0.40 (the smoking-risk item); five of the seven items exceeded the conventional 0.20 minimum, while the contagion item showed negative discrimination. This lower reliability reflects the heterogeneous, multi-domain content of the scale—general awareness, treatability belief, contagion misconception, three distinct modifiable risk factors, and early detection awareness—for which high internal consistency is not theoretically expected, as each item probes a conceptually distinct piece of factual knowledge rather than a single underlying trait [25]. All seven items were retained for continuity with the a priori scoring protocol and to preserve face-validity coverage of key misconceptions; this limitation is discussed transparently in Section 4.5, and full item-level detail is provided in Supplementary Table S4. Participants scoring ≥ 5 were classified as having high knowledge [7].

The complete questionnaire comprised five sections:

- Section 1—Sociodemographic characteristics (9 items).
- Section 2—Health and family cancer history (3 items).
- Section 3—Breast cancer knowledge and awareness (19 items); adapted from [7,21,23,24].
- Section 4—Barriers to mammography screening (10 items); adapted from CHBMS [14,15] plus four Saudi-specific items [7,17,18,23].
- Section 5—Attitudes toward breast cancer screening (6 Likert items); adapted from CHBMS subscales [13,14,15].

### 2.4. Data Collection Procedure

Data collection used two modalities: (i) an electronic questionnaire distributed via the KSU institutional email distribution list and social media platforms, and (ii) paper-based forms administered in person at PHCs, hospitals, and community centers across all 13 regions by trained bilingual (Arabic/English) research assistants who monitored regional quota completion.

### 2.5. Ethical Considerations

The study was approved by the IRB of King Saud University, College of Applied Medical Sciences (IRB approval number: KSU-HE-24-1113; November 2024). All procedures conformed to the Declaration of Helsinki (2013 revision). Written informed consent was obtained from all participants; anonymity was maintained throughout.

### 2.6. Statistical Analysis

Data were analyzed using IBM SPSS Statistics Version 26.0 (IBM Corp., Armonk, NY, USA) and cross-checked in Python (SciPy 1.11/statsmodels 0.14) during revision. Because the composite knowledge score is a bounded, discrete 0–7 index with extensive ties, the Shapiro–Wilk test is, as expected for such variables, significant (W = 0.94, *p* < 0.001), indicating departure from normality; the knowledge score is therefore summarized by median and interquartile range (IQR) in addition to mean ± SD, and group comparisons use the Mann–Whitney U or Kruskal–Wallis test rather than *t*-tests/ANOVA (Table S1). Age-stratified screening rates were generated from cross-tabulation of age groups against screening uptake in SPSS. The primary outcome was any breast cancer screening (any method) in the preceding five years, coded as screened = 1 and not screened = 0; mammography specifically (Q28) and the recommended mammography target subgroup (≥40 years) were analyzed as distinct, explicitly labeled secondary outcomes (Section 3.4 and Section 3.8). Pearson Chi-square (χ^2^) tests examined bivariate associations between each candidate predictor and the primary outcome; Cramér’s V was computed as the effect size. Where a categorical predictor included strata with expected cell counts < 5 (age ≥ 60 years, *n* = 9; income > 20,000 SAR, *n* = 12), the top two categories were merged for the Chi-square test only (yielding ≥ 50 years and ≥15,001 SAR strata; minimum expected count ≥ 22 in both merged tests), while the full unmerged categories are retained in the descriptive tables for transparency. Significant bivariate predictors (*p* < 0.05) plus theoretically grounded CHBMS Barrier predictors [13,14] were entered simultaneously (enter method, selected to evaluate the full CHBMS-specified predictor set [16]) into binary logistic regression, with age group and monthly income entered as ordinal terms (coded 1–5) under the assumption of a monotonic trend in log-odds across categories; this assumption was checked in a dummy-coded sensitivity model (reference categories: 20–29 years and ≤5000 SAR; Supplementary Table S6), which confirmed the direction and significance of all predictors but revealed attenuated, non-significant effects in the smallest extreme strata (age ≥ 60 years and income > 20,000 SAR), consistent with limited power in these cells rather than a violation of the broader trend. As a further sensitivity analysis addressing the distinction between any-screening and mammography-specific uptake, the regression was re-estimated and restricted to women aged ≥40 years (*n* = 136) with mammography uptake as the outcome (Supplementary Table S7); this age-restricted model did not reach overall significance, underscoring that the full-sample associations reported below should not be extrapolated to the mammography-specific, age-eligible question without caveat (see Section 3.8 and Section 4.2). Adjusted odds ratios (ORs) with 95% CIs are reported. All retained predictors are as follows: VIF range: 1.07–2.43 (no multicollinearity concern). Model calibration: Hosmer–Lemeshow test; discrimination: McFadden’s pseudo R^2^. Marital status was excluded from the primary model due to collinearity with age (Spearman ρ = 0.74) and evaluated separately (Table S5). MCAR analysis confirmed non-systematic missingness for Section 5 non-completers (see Section 3.6). Significance threshold: α = 0.05 (two-tailed).

## 3. Results

### 3.1. Sociodemographic Characteristics of Participants

A total of 426 females were enrolled (100% of the predetermined quota). The 20–29 age group was most prevalent (*n* = 182; 42.7%); women aged ≥40 years—the primary mammography target group—constituted 31.9% (*n* = 136). Single (42.0%) and married (39.9%) participants accounted for 81.9% of the sample. Saudi nationals: 91.3%; bachelor’s degree holders: 43.9%; employees: 30.3%; students: 28.6%; monthly household income ≤ 5000 SAR: 38.0%; and urban residents: 60.8%. A total of 84.8% lived within 10 km of the nearest PHC (Table 1). To orient the denominators used throughout this section, all percentages are of the full analytic sample (*N* = 426) unless otherwise specified; the age-eligible mammography subgroup is *n* = 136 (≥40 years); the family cancer history subgroup is *n* = 254, with *n* = 227 providing a valid response to the breast-cancer-specific follow-up item (Section 3.2); and the physician-consultation item denominator is *n* = 326.

### 3.2. Health Status and Family Cancer History

More than half (59.6%) reported a family history of any cancer—a broader category than first-degree relatives, since the underlying Arabic item asks whether anyone in the participant’s family has ever been diagnosed with cancer; this extended-family framing, rather than selection bias, most plausibly explains the high endorsement rate. The breast-cancer-specific follow-up item was intended to apply only to the 254 women who reported a family history of any cancer; however, because the questionnaire (a Google Form) did not skip-logic-gate this item, 113 women who had reported no family history of any cancer also answered it, while 27 of the 254 eligible women left it blank. Restricting the denominator to the 254 women for whom the item was logically applicable, 227 (89.4%) provided a valid response, of whom 147 (64.8% of 227; 57.9% of the full 254) confirmed a family history of breast cancer specifically. This denominator reconciliation, and the underlying instrument-design limitation, are discussed further in Section 4.5. Of the 326 who responded to the physician-consultation question, 55.8% (*n* = 182) had consulted a physician, while 44.2% had not (Table 2).

### 3.3. Breast Cancer Knowledge and Awareness

The mean composite knowledge score was 4.51 ± 1.52 (range 0–7; median = 5; KR-20 = 0.45; item-total r: −0.11 to 0.40); 54.0% achieved high knowledge (≥5/7). General awareness was high (86.2%); 88.7% recognized the importance of early detection. Specific knowledge was more limited: only 42.3% correctly identified obesity as a risk factor; 23.5% incorrectly believed breast cancer to be contagious; 25.8% were uncertain whether it was treatable; and only 52.1% correctly identified age 40 as the mammography initiation age. Social media was the primary information source for 47.2% versus physicians for 21.1%. Among Q24’s six response options, ‘family or friends’ and ‘television or print media’ were each selected by <0.5% and were consolidated into ‘other’ (Table 3).

### 3.4. Age-Stratified Breast Cancer Screening Rates

Screening rates were derived from cross-tabulation of age group against screening uptake in SPSS Version 26.0 (Table 4). Among women aged ≥40 (*n* = 136), 70.6% had undergone any breast cancer screening and 52.2% mammography in the preceding five years. This 52.2% figure is the most policy-relevant single estimate in this study, as it describes uptake within the age-eligible target group; for context, the WHO GBCI Pillar 1 indicator concerns the proportion of invasive cancers diagnosed at stage I/II rather than mammography coverage and so is not directly comparable [10]. Among women aged <40 (*n* = 290), 31.4% had undergone any screening, and 20.3% mammography. These findings are consistent with a 2025 Saudi cross-sectional survey (*n* = 487) reporting 53.2% underwent mammography among women aged ≥40 versus 12.3% among those aged <40 [26].

### 3.5. Barriers to Mammography Screening

Although 71.6% acknowledged the importance of mammography, barriers were prevalent across all domains (Table 5). Fear of a cancer diagnosis (83.6%; CHBMS Barriers) was most prevalent, followed by belief in incurability (76.3%), radiation concern (73.2%; CHBMS), and pain anxiety (72.3%; CHBMS). Procedural unfamiliarity (56.3%) and embarrassment (54.5%) are also CHBMS Barriers items. Saudi-specific structural barriers included transportation difficulties (47.9%) and family discouragement (34.5%).

### 3.6. Attitudes Toward Breast Cancer Screening and Missing Data Analysis

Seventy-one participants (16.7%) did not complete Section 5 and were excluded from attitude analyses (*n* = 355). MCAR analysis using independent-samples t-tests and Pearson’s Chi-square found non-significant differences between completers and non-completers in age group (χ^2^(4) = 4.21, *p* = 0.378), educational level (χ^2^(5) = 3.87, *p* = 0.568), screening uptake (χ^2^(1) = 1.94, *p* = 0.163), and knowledge score (t = 0.89, *p* = 0.374), supporting the MCAR assumption. Overall attitude responses were strongly positive: 93.2% agreed or strongly agreed that all women should undergo regular breast cancer screening (mean 4.52 ± 0.65; CHBMS Benefits). Negatively worded barrier items were correctly rejected by 78.3% and 77.2% (Table 6).

### 3.7. Bivariate Analysis

All seven tested variables demonstrated statistically significant associations with breast cancer screening uptake (Table 7). Degrees of freedom are corrected to match Table 1 category counts (df = k − 1), except for age group and monthly income, for which the top two categories are merged prior to testing (≥50 years; ≥15,001 SAR) because the unmerged ≥60-years and >20,000-SAR strata have expected cell counts below 5 (Section 2.6); the full unmerged breakdown remains available descriptively in Table 1 and Table 4. Using the merged categories, age group (χ^2^(3) = 87.12, V = 0.452, *p* < 0.001) and monthly income (χ^2^(3) = 82.88, V = 0.441, *p* < 0.001) show the largest effects, with both tests now satisfying the minimum expected count assumption (minimum expected count ≥22 in both). Place of residence also reached significance (χ^2^(2) = 7.63, V = 0.134, *p* = 0.022), with urban women demonstrating substantially higher screening rates than rural women, consistent with the regional variation documented in Supplementary Table S2.

### 3.8. Binary Logistic Regression

The eight variables entered simultaneously into the model were: age group, monthly household income, high knowledge score (≥5), and five CHBMS-grounded barrier items (fear of diagnosis, radiation concern, pain anxiety, transportation barrier, and family discouragement); age and income were entered as ordinal terms (Section 2.6). The overall model was significant (χ^2^(8) = 188.96, *p* < 0.001; McFadden’s pseudo R^2^ = 0.323; Hosmer–Lemeshow: χ^2^(8) = 8.42, *p* = 0.394; VIF range: 1.07–2.43). Older age (OR = 1.52; 95% CI: 1.21–1.92; *p* < 0.001) and higher monthly income (OR = 1.57; 95% CI: 1.25–1.99; *p* < 0.001) were independent positive predictors. Transportation barriers (OR = 3.39; 95% CI: 1.95–5.89; *p* < 0.001) and family discouragement (OR = 3.03; 95% CI: 1.72–5.34; *p* < 0.001) were significant contextual predictors. This positive direction is counterintuitive on its face and is therefore checked against the raw data using explicit screened = 1/not screened = 0 coding; the result is reproduced exactly, ruling out a coding artifact (Section 2.6). Positive OR directions possibly reflect an experiential-reporting mechanism [27] whereby women who have already attempted or completed screening have direct exposure to transport logistics and family reactions; this interpretation is offered as a hypothesis rather than an established mechanism, as the cross-sectional design cannot establish temporal order [28] (see Section 4.3). The three CHBMS Barrier constructs and the knowledge score (OR = 1.46, *p* = 0.141) were non-significant after multivariate adjustment—consistent with HBM theory [16] and meta-analytic evidence [29] that knowledge provision alone is insufficient when barriers are high. A dummy-coded sensitivity model and an age-restricted mammography-specific sensitivity model are reported in Supplementary Tables S6 and S7, respectively, and discussed in Section 4.2 (Table 8).

## 4. Discussion

This geographically broad cross-sectional study, covering all 13 administrative regions of Saudi Arabia, provides one of the most geographically comprehensive KAP analyses of breast cancer screening among females in Saudi Arabia to date. Consistent with the interpretive hierarchy established in Section 3.4, the pooled mammography rate of 30.5% describes screening experience across the full surveyed population and is demographically diluted by the predominance of participants aged <40 (68.1%), who fall outside the recommended screening age; the more policy-relevant estimate is the 52.2% mammography uptake among the age-eligible subgroup (≥40 years, *n* = 136). The WHO GBCI Pillar 1 indicator concerns the proportion of cancers diagnosed at stage I/II, not mammography coverage, and so cannot be used as a direct benchmark for either figure [10]. Interpreted through the CHBMS framework [13,14,15] and HBM [16], this knowledge–practice gap reflects CHBMS-identified barriers substantially outweighing perceived benefits.

### 4.1. Knowledge and Awareness: Gains and Persistent Gaps

The 86.2% general awareness rate exceeds the pooled estimate of 66.3% from AlRajhi et al.’s systematic review [19] and is consistent with recent Saudi data [30,31]. Social media now constitutes the primary information source for 47.2% of participants versus physicians (21.1%). A 2025 MENA literature review confirmed comparable patterns of broad awareness coexisting with marked specific knowledge gaps [20], and a broader study (*n* = 2681) found fewer than 54% of Arab women could identify five or more breast cancer symptoms [32]. Specific knowledge deficits were clinically consequential: only 42.3% identified obesity as a risk factor [23,33,34]; 23.5% believed breast cancer to be contagious [20,35]; and only 52.1% correctly identified age 40 as the mammography initiation age. The non-significance of the knowledge score in the logistic regression (OR = 1.46, *p* = 0.141) is consistent with HBM theory [16] and meta-analytic evidence [29], confirming that knowledge is necessary but insufficient when CHBMS Barriers are high.

### 4.2. Screening Practices: Interpreting the Overall and Age-Stratified Rates

Among women aged ≥40 (*n* = 136), mammography uptake was 52.2%; among women aged <40 (*n* = 290), the rate was 20.3%. A 2025 Saudi cross-sectional survey (*n* = 487) reported convergent findings: 53.2% among ≥40 versus 12.3% among <40 [26]. Alshamsan et al. (2024) found that 23.6% of women aged 40–69 attending the BCED program in Qassim declined a free mammogram [36]. Consistent with El Bcheraoui et al. [12], Gouse et al. [37], and Albanghali et al. [38], these findings confirm that the free-service model alone is insufficient to achieve high mammography uptake without targeted intervention.

### 4.3. CHBMS Barrier Profile and Saudi-Specific Additions

Fear of a cancer diagnosis (83.6%)—a core CHBMS Barriers construct validated in Arabic by Mikhail and Petro-Nustas [15]—was the dominant barrier, consistent with findings across Saudi Arabia [18,36,39], the MENA region [20,32], and Muslim-majority countries globally [40]. Stage-specific survival data underscore the consequence of this fear-avoidance dynamic: this convergence across culturally and economically diverse settings—from Gulf Cooperation Council countries to broader MENA populations and other Muslim-majority contexts outside the region [40]—suggests that fear of a cancer diagnosis functions as a relatively culture-general psychological barrier to screening rather than one specific to the Saudi context, even though the structural and family-related barriers reported here (Section 3.5 and Section 4.4) are more clearly shaped by local social and health-system factors. Five-year survival exceeds 99% for localized (stage I) breast cancer compared with approximately 29% for metastatic disease [2]—a 70-percentage-point differential attributable almost entirely to stage at diagnosis.

Radiation concern (73.2%) and pain anxiety (72.3%)—both CHBMS Barrier items [14]—are correctable informational deficits. Modern full-field digital mammography typically delivers a mean glandular dose on the order of 1–3 mGy per view, depending on breast thickness, density, and acquisition technique, well within international (EU/IAEA) reference limits of <2.5 mGy for a standard breast; dosimetric modeling indicates that the benefit of detecting cancer substantially outweighs this radiation risk across realistic screening scenarios [41]. Standardized patient information targeting these CHBMS constructs, consistent with model-based intervention evidence [29], could efficiently reduce barriers at scale. The positive ORs for transportation barriers and family discouragement are counterintuitive at first glance, since such barriers would be expected to reduce rather than increase screening participation; this pattern is empirically reproduced from the raw data using explicit screened = 1/not screened = 0 coding (Section 2.6), ruling out a simple coding artifact. A plausible explanation, consistent with an experiential-reporting mechanism [27], is that these associations may reflect experiential reporting among women who have already attempted or completed screening—who have direct exposure to transport logistics and family reactions around the screening visit itself—rather than these barriers acting only as antecedent deterrents [28]. However, directionality cannot be inferred from the present cross-sectional design, and this interpretation should be treated as a hypothesis for future longitudinal testing rather than an established mechanism. Regional variation—from Qassim: 20.0% to Northern Borders: 76.9%—underscores the urgency of mobile mammography expansion and organized invitation systems in low-uptake regions.

### 4.4. Socioeconomic Determinants and Geographic Access Inequities

Older age (OR = 1.52) and higher income (OR = 1.57) were independent positive predictors, consistent with the global literature [21,28] and HBM’s health motivation and self-efficacy constructs [16]. Mammography uptake was significantly more concentrated among wealthier and better-educated Saudi women (income concentration index = 0.163) [5], confirming that indirect costs—transportation, childcare, and lost wages—disproportionately burden lower-income women even where services are nominally free [5]. The significant association between places of residence (χ^2^(2) = 7.63, V = 0.134, *p* = 0.022) confirms that urban women had substantially higher screening rates than rural counterparts, aligning with regional variation in Supplementary Table S2 and supporting mobile mammography deployment in rural Saudi Arabia [20]. More broadly, the fact that all seven variables tested in the bivariate analysis (Table 7) reached significance is consistent with these sociodemographic factors being substantially correlated with one another rather than fully independent: age is associated with marital status, income, and education in this sample, and these shared associations likely inflate the number of bivariate associations relative to what survives multivariable adjustment, where only age, income, and two barrier variables remained independently significant (Section 3.8, Table 8).

### 4.5. Strengths and Limitations

Principal strengths include geographic breadth across all 13 Saudi administrative regions; use of the Arabic-validated CHBMS [13,14,15]; CVI-assessed content validity (mean = 0.91); and a full analytical robustness program comprising Hosmer–Lemeshow calibration, VIF verification, MCAR analysis, and two sensitivity models (dummy-coded and age-restricted; Supplementary Tables S6 and S7).

Several limitations should be weighed when interpreting these findings. Most importantly, the quota-based, non-probability recruitment strategy over-represents younger, educated, and urban women and precludes nationally representative inference. Other limitations include the cross-sectional design, which limits causal interpretation; self-reported screening status, subject to social desirability and recall bias; a skip-logic gap in the family cancer history item requiring post hoc denominator correction; internal inconsistency in self-reported screening responses for 9.4% of participants; absence of self-efficacy items—a key HBM construct that may partly explain unexplained model variance; and lower-than-expected knowledge-scale reliability (KR-20 = 0.45), reflecting the scale’s heterogeneous multi-domain content. Future studies should use probability sampling, the full CHBMS with self-efficacy subscales, and objectively verified screening records.

## 5. Conclusions

This geographically broad study, covering all 13 administrative regions of Saudi Arabia, documented a significant knowledge–practice gap in breast cancer screening among females in Saudi Arabia. The pooled mammography rate of 30.5% reflects the predominance of participants below the recommended screening age; among eligible women aged ≥40, the more policy-relevant rate of 52.2% remained below the commonly cited 60% mammography-coverage aspiration referenced in the regional literature, noting that the WHO GBCI’s own Pillar 1 indicator concerns stage at diagnosis rather than screening coverage. CHBMS Barrier constructs—fear of diagnosis, radiation and pain anxiety, and embarrassment—were highly prevalent, compounded by Saudi-specific barriers including belief in incurability, family discouragement, and transportation difficulties. Older age and higher income were independently associated with higher screening uptake; geographic access constraints—evidenced by significant associations for both PHC distance and place of residence—exacerbate existing socioeconomic inequities. As this is a cross-sectional, self-reported survey, these associations should be interpreted as statistical correlates rather than causal determinants. The non-significance of the composite knowledge score as a predictor in the adjusted model is consistent with HBM’s core proposition: knowledge provision alone is insufficient to change health behavior when perceived barriers are high.

These findings, while not derived from an intervention trial, suggest several implications for a multi-level public health response, to be evaluated through future intervention research: (i) barrier-targeted psychoeducational campaigns correcting misconceptions about treatability, radiation safety, and universal screening need, and communicating stage-specific survival contrasts [2]; (ii) engagement of family members, including spouses, brothers, and fathers who influence women’s health-seeking decisions, given the significant association observed for family discouragement; (iii) mobile mammography expansion and organized invitation systems in Qassim (20.0%), Riyadh (28.7%), and other low-uptake regions; (iv) breast awareness education and prompt symptom-reporting counseling integrated into all PHC encounters, beginning with the 20–29 cohort, consistent with the WHO’s current emphasis on awareness and timely reporting over formal self-examination training [10]; and (v) regulation of MOH-endorsed digital health content to counteract social media misinformation [42]. Because self-efficacy (confidence in one’s ability to act on screening intentions) is not measured in the present CHBMS adaptation, it remains a plausible mediating factor between knowledge, barriers, and uptake that this study could not directly test; longitudinal studies using the full CHBMS [13,14,15] with self-efficacy subscales and objectively verified screening records constitute the most pressing research priority.

## Figures and Tables

**Table 1:** Sociodemographic characteristics of study participants (*n* = 426).

Characteristic	*n*	%
Age group (years)		
20–29	182	42.7
30–39	108	25.4
40–49	70	16.4
50–59	57	13.4
≥60	9	2.1
Marital status		
Single	179	42.0
Married	170	39.9
Divorced	64	15.0
Widowed	13	3.1
Nationality		
Saudi	389	91.3
Non-Saudi	37	8.7
Educational level		
Primary or below	9	2.1
Intermediate	33	7.7
High school	74	17.4
Diploma	82	19.2
Bachelor’s degree	187	43.9
Postgraduate	40	9.4
Occupation		
Student	122	28.6
Employee	129	30.3
Self-employed	63	14.8
Retired	57	13.4
Job seeker/unemployed	55	12.9
Monthly household income (SAR)		
≤5000	162	38.0
5001–10,000	117	27.5
10,001–15,000	97	22.8
15,001–20,000	38	8.9
>20,000	12	2.8
Place of residence		
City	259	60.8
Governorate/town	120	28.2
Village/rural	47	11.0
Distance to nearest PHC		
<5 km	178	41.8
6–10 km	183	43.0
11–15 km	54	12.7
≥16 km	11	2.6

Note: PHC, primary healthcare center; SAR, Saudi Arabian Riyal. Women aged ≥40 (mammography target group): *n* = 136 (31.9%). Percentages may not total 100.0% due to rounding.

**Table 2:** Health status and family cancer history (*n* = 426).

Variable	*n*	%
Family history of any cancer		
Yes	254	59.6
No	172	40.4
If yes—specifically breast cancer? (*n* = 227 of the 254 women reported any family cancer history)		
Yes	147	64.8
No	80	35.2
Have consulted physician due to breast cancer family history (*n* = 326 respondents)		
Yes	182	55.8
No	144	44.2

Note: The breast-cancer-specific item is not skip-logic-gated in the survey instrument; the denominator (*n* = 227) is restricted to the 254 women reporting any family cancer history who provided a valid response to this item (Section 3.2). The physician-consultation denominator (*n* = 326) reflects actual respondent counts for that sub-question.

**Table 3:** Breast cancer knowledge, awareness, and screening practices (*n* = 426).

Item	Response	*n*	%
**Disease knowledge**			
Aware of breast cancer	Yes	367	86.2
	No	59	13.8
Breast cancer is treatable	Yes	229	53.8
	No	87	20.4
	Not sure	110	25.8
Breast cancer is contagious *	Yes (incorrect)	100	23.5
	No (correct)	288	67.6
	Not sure	38	8.9
**Modifiable risk factor knowledge**			
Obesity increases risk	Yes	180	42.3
	No	113	26.5
	Not sure	133	31.2
Physical inactivity increases risk	Yes	223	52.3
	No	70	16.4
	Not sure	133	31.2
Smoking increases risk	Yes	255	59.9
	No	68	15.9
	Not sure	103	24.2
**Early detection knowledge**			
Aware of early detection importance	Yes	379	88.7
	No	47	11.3
Early detection improves outcomes	Yes	338	79.2
	No	60	14.1
	Not sure	28	6.6
**Awareness engagement**			
Attended BC awareness campaign	Yes—beneficial	255	59.9
	Yes—not beneficial	37	8.6
	No	134	31.5
Received BC awareness at PHC	Yes	260	61.0
	No	166	39.0
Primary information source (Q24) **	Social media	201	47.2
	Awareness campaigns	128	30.0
	Physician	90	21.1
	Other	7	1.6
**Screening practices**			
Heard of mammography	Yes	286	67.1
	No	140	32.9
Screened in past 5 years (any method)	Yes	187	43.9
	No	239	56.1
Underwent mammography	Yes	130	30.5
	No	261	61.3
	Alternative method only	35	8.2
**Mammography knowledge**			
Correct target age (≥40 years)	Yes (correct)	222	52.1
	No/Not sure	204	47.9
Mammography radiation is safe	Yes	182	42.7
	No	50	11.7
	Not sure	194	45.5

Note: BC, breast cancer; PHC, primary healthcare center. * Correct response: ‘No’. ** Q24 offered six options; ‘family or friends’ and ‘television or print media’ were each selected by <0.5% and consolidated into ‘other’. Knowledge score: Mean = 4.51 ± 1.52/7; KR-20 = 0.45; item-total r: −0.11 to 0.40; and 54.0% had high knowledge (≥5). Mammography uptake figures reflect corrected values (Section 3.2); 40 of 426 (9.4%) respondents gave internally inconsistent answers between the any-screening and mammography-specific items, which were disclosed as instrument limitation (Section 4.5). Percentages may not sum to 100% due to rounding.

**Table 4:** Age-stratified breast cancer screening rates (*n* = 426).

Age Group	*n*	Any Screening *n* (%)	Mammography *n* (%)	Mammography Target Age?
20–29 years	182	35 (19.2%)	27 (14.8%)	No
30–39 years	108	56 (51.9%)	32 (29.6%)	No
40–49 years	70	50 (71.4%)	39 (55.7%)	Yes
50–59 years	57	42 (73.7%)	31 (54.4%)	Yes
≥60 years	9	4 (44.4%)	1 (11.1%)	Yes
Total aged ≥40 (recommended group)	136	96 (70.6%)	71 (52.2%)	—
Total aged <40	290	91 (31.4%)	59 (20.3%)	—
Overall total	426	187 (43.9%)	130 (30.5%)	—

Note: Derived from cross-tabulation in SPSS Version 26.0. MOH mammography target age: ≥40 years [11]. The WHO GBCI Pillar 1 target (≥60% of invasive cancers diagnosed at stage I/II [10]) is a stage-at-diagnosis indicator, not a mammography-coverage benchmark, and is not directly comparable to the uptake figures above. The pooled rate (30.5%) reflects the inclusion of 68.1% of participants who are below the recommended screening age.

**Table 5:** Prevalence of self-reported barriers to mammography screening (*n* = 426).

Barrier Item	Domain/Source	Yes—*n* (%)	No—*n* (%) *
CHBMS Barrier items [13,14,15]			
Fear of cancer diagnosis	CHBMS Barriers	356 (83.6%)	70 (16.4%)
Radiation exposure concern	CHBMS Barriers	312 (73.2%)	114 (26.8%)
Pain/procedural discomfort	CHBMS Barriers	308 (72.3%)	118 (27.7%)
Embarrassment about mammogram	CHBMS Barriers	232 (54.5%)	194 (45.5%)
Unfamiliar with screening procedure	CHBMS Barriers	240 (56.3%)	186 (43.7%)
Does not believe screening is important	CHBMS Benefits (inverse) †	121 (28.4%)	305 (71.6%)
Saudi-specific barrier items			
Believes breast cancer has no treatment	[23]	325 (76.3%)	101 (23.7%)
Believes screening unnecessary without family history	[7]	185 (43.4%)	241 (56.6%)
Transportation difficulties	[18]	204 (47.9%)	222 (52.1%)
Family discouragement	[17]	147 (34.5%)	279 (65.5%)

Note: CHBMS, Champion’s Health Belief Model Scale [13,14]; Arabic validation: [15]. * ‘Not sure’ responses recorded as not endorsing; this conservative choice treats expressed uncertainty as non-endorsement for analytic purposes, which may understate true ambivalence but avoids overstating positive attitudes from equivocal responses. † ‘CHBMS Benefits (inverse)’ denotes low perceived benefit—the inverse of the CHBMS Benefits subscale orientation. Percentages may not sum to 100% due to rounding.

**Table 6:** CHBMS-adapted attitude items—five-point Likert scale (*n* = 355).

Statement (CHBMS Domain)	*n*	Mean ± SD	SA (%)	A (%)	U (%)	D (%)	SD (%)
All women should undergo regular BC screening (Benefits)	355	4.52 ± 0.65	60.0	33.2	5.9	0.8	0.0
Screening enables early detection of BC (Benefits)	355	4.60 ± 0.64	68.7	23.1	7.9	0.3	0.0
Early detection prevents complications (Benefits)	355	4.16 ± 0.97	44.8	35.5	12.1	5.9	1.7
Survival does NOT depend on early detection ** (Barriers)	355	4.07 ± 1.09	5.9	2.8	14.1	33.8	43.4
BSE does NOT help detect BC ** (Barriers)	355	4.52 ± 0.62	0.3	1.7	19.7	28.2	50.1
Women with BC family history need not worry ** (Barriers)	355	3.71 ± 1.40	8.5	14.1	11.3	23.1	43.1

Note: Items adapted from CHBMS Benefits and Barriers subscales [13,14]; Arabic validation: [15]. SA, Strongly Agree; A, Agree; U, Undecided; D, Disagree; SD, Strongly Disagree; BC, breast cancer; BSE, breast self-examination. ** Negatively worded: D + SD = correct positive attitude. Cronbach’s α = 0.74. *n* = 355; MCAR *p* > 0.05 for all comparisons (SPSS output available on request).

**Table 7:** Chi-square analysis: bivariate associations with breast cancer screening uptake (*n* = 426).

Variable	χ^2^ Statistic	df a	Cramér’s V	*p*-Value	Significance
Age group	90.31	4	0.460	<0.001	***
Monthly household income	94.42	4	0.471	<0.001	***
Marital status	63.14	3	0.385	<0.001	***
Family history of any cancer	32.59	1	0.277	<0.001	***
Educational level	22.53	5	0.230	<0.001	***
Distance to nearest PHC	13.18	3	0.176	0.004	**
Place of residence	7.63	2	0.134	0.022	*

Note: a Degrees of freedom corrected to match category counts in Table 1 (df = k − 1; binary outcome). For age and monthly income, the top two categories were merged (≥50 years; ≥15,001 SAR) to satisfy the minimum expected cell count assumption (Section 2.6); the unmerged breakdown is shown descriptively in Table 1 and Table 4. PHC, primary healthcare center; V, Cramér’s V. * *p* < 0.05; ** *p* < 0.01; *** *p* < 0.001.

**Table 8:** Binary logistic regression: independent predictors of breast cancer screening uptake in the preceding five years (*n* = 426).

Predictor Variable (Source)	β	SE	Wald χ^2^	OR	95% CI	*p*-Value
Age group	0.420	0.117	12.87	1.52	1.21–1.92	<0.001 ***
Monthly household income	0.454	0.119	14.58	1.57	1.25–1.99	<0.001 ***
High knowledge score ≥ 5	0.378	0.257	2.16	1.46	0.88–2.41	0.141
Fear of diagnosis (CHBMS Barriers)	0.254	0.372	0.47	1.29	0.62–2.68	0.494
Radiation concern (CHBMS Barriers)	0.318	0.336	0.90	1.37	0.71–2.66	0.344
Pain anxiety (CHBMS Barriers)	0.438	0.321	1.86	1.55	0.83–2.91	0.172
Transportation barrier	1.219	0.282	18.65	3.39	1.95–5.89	<0.001 ***
Family discouragement	1.107	0.290	14.57	3.03	1.72–5.34	<0.001 ***

Note: β, regression coefficient; SE, standard error; OR, adjusted odds ratio; CI, confidence interval. Age group and monthly income entered as ordinal terms (1–5); a dummy-coded sensitivity model is reported in Supplementary Table S6, and an age-restricted (≥40 years) mammography-specific sensitivity model in Supplementary Table S7. VIF range: 1.07–2.43. Model: χ^2^(8) = 188.96, *p* < 0.001; McFadden’s pseudo R^2^ = 0.323; Hosmer–Lemeshow χ^2^(8) = 8.42, *p* = 0.394. *** *p* < 0.001.

## Data Availability

The anonymized data supporting reported results are available from the corresponding author (nalruwaili@ksu.edu.sa) upon reasonable request, subject to participant privacy restrictions.

## Supplementary Materials

The following supporting information can be downloaded at https://www.mdpi.com/article/10.3390/healthcare14132003/s1, Supplementary File S1: Study questionnaire (English version); Supplementary File S2: STROBE checklist for cross-sectional studies [43]; Supplementary File S3: Supplementary statistical tables (Table S1. Composite knowledge score by age group (*n* = 426); Table S2. Breast cancer screening uptake (any method, preceding 5 years) by administrative region (*n* = 426); Table S3. Breast cancer screening uptake by marital status (*n* = 426); Table S4. Item-level frequency distribution and item-total correlations for composite knowledge score (*n* = 426); Table S5. Sensitivity analysis: binary logistic regression including marital status (*n* = 426); Table S6. Sensitivity analysis: binary logistic regression with age and monthly income entered as dummy-coded categorical variables (reference: 20–29 years; ≤5000 SAR) (*n* = 426); Table S7. Sensitivity analysis: binary logistic regression for mammography-specific uptake restricted to age-eligible women (≥40 years) (*n* = 136). Overall model not statistically significant (likelihood-ratio χ^2^(8) = 16.51, *p* = 0.208; pseudo R^2^ = 0.058); Figure S1. Participant Recruitment and Inclusion Flow Diagram).

## Abbreviations

The following abbreviations are used in this manuscript:

BC	Breast cancer
BCAM	Breast Cancer Awareness Measure
BSE	Breast self-examination
CBE	Clinical breast examination
CHBMS	Champion’s Health Belief Model Scale
CI	Confidence interval
CVI	Content validity index
HBM	Health Belief Model
IRB	Institutional Review Board
KAP	Knowledge, attitudes, and practices
MCAR	Missing completely at random
MOH	Ministry of Health
OR	Odds ratio
PHC	Primary healthcare center
SAR	Saudi Arabian Riyal
SD	Standard deviation
STROBE	Strengthening the Reporting of Observational Studies in Epidemiology
VIF	Variance inflation factor
WHO	World Health Organization
WHO GBCI	WHO Global Breast Cancer Initiative
