# Supplementary material for: Knowledge, Attitudes, and Practices Regarding Breast Cancer Screening Among Females in Saudi Arabia
Source: Healthcare (Basel). 2026 Jul 6;14(13):2003. doi: 10.3390/healthcare14132003 (PMC13362469; doi:10.3390/healthcare14132003)
Supplement: Supplementary file 1 [file healthcare-14-02003-s001.zip › S1_Study Questionnaire_Healthcare.pdf]

Supplementary File S1

Study Questionnaire — English Version

Knowledge, Attitudes, and Practices Regarding Breast Cancer Screening Among Females in Saudi Arabia

Nawaf W. Alruwaili[1,*], Abdullah Mohammed Alfehaid[1,2], Khaled Abdullah Shafi Al-Toum[1,3], Aljazi Bin Zarah[1] and Nora Alafif[1]

[1] Department of Community Health Sciences, College of Applied Medical Sciences, King Saud University, Riyadh 11433, Saudi Arabia; 445911983@student.ksu.edu.sa (A.M.A.); kaltoum@moh.gov.sa (K.A.S.A.-T.); abinzaraah@ksu.edu.sa (A.B.Z.); nalafeef@ksu.edu.sa (N.A.)

[2] Public Health Authority, Riyadh 13352, Saudi Arabia

[3] Ministry of Health, Riyadh 12822, Saudi Arabia

Instructions: This questionnaire is anonymous and confidential. Responses are used solely for academic research. Please answer all items honestly by placing a tick (✓) in the appropriate box. Estimated completion time: 8–10 minutes.

Section I — Personal Information

1. Age group:

- ☐ 20–29 years
- ☐ 30–39 years
- ☐ 40–49 years
- ☐ 50–59 years
- ☐ 60 years and above

2. Marital status:

- ☐ Single
- ☐ Married
- ☐ Divorced
- ☐ Widowed

3. Nationality:

- ☐ Saudi
- ☐ Non-Saudi

4. Educational level:

- ☐ Primary school or below
- ☐ Intermediate school
- ☐ High school
- ☐ Diploma
- ☐ Bachelor's degree
- ☐ Postgraduate (Master's or PhD)

5. Occupation:

- ☐ Student
- ☐ Employee (government or private sector)
- ☐ Self-employed
- ☐ Retired
- ☐ Job seeker/unemployed

6. Monthly household income (SAR):

- ☐ 5,000 SAR or less
- ☐ 5,001–10,000 SAR
- ☐ 10,001–15,000 SAR
- ☐ 15,001–20,000 SAR
- ☐ 20,000 SAR and above

7. Region of residence:

- ☐ Riyadh
- ☐ Eastern Province
- ☐ Makkah
- ☐ Madinah
- ☐ Qassim
- ☐ Tabuk
- ☐ Najran
- ☐ Ha'il
- ☐ Northern Borders
- ☐ Al-Jouf
- ☐ Asir
- ☐ Jazan
- ☐ Al-Baha
- ☐ Other

8. Place of residence:

- ☐ City
- ☐ Governorate/town
- ☐ Village

9. Distance from home to nearest primary healthcare center (PHC):

- ☐ Less than 5 km
- ☐ 6–10 km
- ☐ 11–15 km
- ☐ 16 km or more

Section II — Health Status and Family Cancer History

10. Has any member of your family ever been diagnosed with cancer (any type)?

☐ Yes
☐ No

11. If yes — was it specifically breast cancer?
☐ Yes
☐ No
☐ Not applicable

12. If you have a family history of breast cancer, do you consult a physician or visit a specialist center?
☐ Yes
☐ No
☐ Not applicable

Section III — Breast Cancer Knowledge and Awareness

13. Are you aware of breast cancer as a disease?
☐ Yes
☐ No

14. Is there an effective treatment for breast cancer?
☐ Yes
☐ No
☐ I don't know

15. Is breast cancer a contagious (transmissible) disease?
☐ Yes
☐ No
☐ I don't know
(Note: Correct response: No — breast cancer is not contagious.)

16. Does obesity (being overweight) increase the risk of developing breast cancer?
☐ Yes
☐ No
☐ I don't know

17. Does physical inactivity (lack of exercise) increase the risk of developing breast cancer?
☐ Yes
☐ No
☐ I don't know

18. Does smoking increase the risk of developing breast cancer?
☐ Yes
☐ No
☐ I don't know

19. Are you aware of the importance of early breast cancer screening?

☐ Yes

☐ No

20. Does early detection of breast cancer improve clinical outcomes (e.g., breast preservation, improved survival)?

☐ Yes

☐ No

☐ I don't know

21. Have you ever attended a breast cancer awareness campaign or health activity?

☐ Yes, and I found it beneficial

☐ Yes, but I did not benefit

☐ No

22. Have you received sufficient breast cancer awareness information at a PHC?

☐ Yes

☐ No

23. If yes (Q22) — was the information provided in a language you could easily understand?

☐ Yes

☐ No

☐ Not applicable

24. Primary source of information about breast cancer (select ONE only):

☐ Physician/healthcare provider

☐ Social media (e.g., Twitter/X, Instagram, TikTok)

☐ Awareness campaigns or health fairs

☐ Family or friends

☐ Television or print media

☐ Other

25. Have you heard about breast cancer screening using mammography (a specialized X-ray of the breast)?

☐ Yes

☐ No

26. In the past five years, have you undergone any form of breast cancer screening?

☐ Yes

☐ No

27. Have you specifically undergone mammography screening?

☐ Yes

☐ No

☐ I have been screened using a different method

28. If yes (Q27) — how frequently do you undergo mammography?

- ☐ Every year
- ☐ Every two years
- ☐ Every three or more years
- ☐ I have only done it once/I don't know

29. Approximately how long does a standard mammography examination take?

- ☐ 10 minutes
- ☐ 20 minutes
- ☐ 30 minutes
- ☐ I don't know

30. According to current clinical guidelines, from what age is routine mammography screening generally recommended?

- ☐ Age 20
- ☐ Age 30
- ☐ Age 40 and above (correct)
- ☐ I don't know

31. Is the radiation dose used in mammography considered safe at recommended screening intervals?

- ☐ Yes
- ☐ No
- ☐ I don't know

Section IV — Barriers to Mammography Screening

32. Do you believe that mammography screening for breast cancer is important?

- ☐ Yes
- ☐ No
- ☐ I don't know

33. Are you concerned about radiation exposure during a mammogram?

- ☐ Yes
- ☐ No

34. Are you worried about pain or discomfort associated with the mammography procedure?

- ☐ Yes
- ☐ No

35. Are you afraid of being diagnosed with breast cancer?

- ☐ Yes
- ☐ No

36. Has a family member ever discouraged you from undergoing breast cancer screening?

☐ Yes
☐ No
☐ I don't know

37. Do you believe that early breast cancer screening is unnecessary if you have no family history of the disease?

☐ Yes
☐ No
☐ I don't know

38. Do you face difficulties with transportation in accessing breast cancer screening services?

☐ Yes
☐ No

39. Do you feel embarrassed or uncomfortable about undergoing a mammogram?

☐ Yes
☐ No

40. Are you afraid of not knowing what to do or how to prepare for a breast cancer screening examination?

☐ Yes
☐ No

41. Are you concerned that breast cancer may not have an effective treatment?

☐ Yes
☐ No

Section V — Attitudes Toward Breast Cancer Screening

Instructions: Please indicate your level of agreement with each statement using the scale: 1 = Strongly Disagree 2 = Disagree 3 = Undecided 4 = Agree 5 = Strongly Agree

Statement	1 SD	2 D	3 U	4 A	5 SA
All women should undergo regular breast cancer screening regardless of symptoms.	☐	☐	☐	☐	☐
Breast cancer screening is important because it enables early detection.	☐	☐	☐	☐	☐
Early detection of breast cancer helps prevent further complications and saves lives.	☐	☐	☐	☐	☐
Breast cancer survival does NOT depend on early detection. *	☐	☐	☐	☐	☐
Breast self-examination does NOT help in detecting breast cancer. *	☐	☐	☐	☐	☐

Statement	1 SD	2 D	3 U	4 A	5 SA
Women with a family history of breast cancer do NOT need to worry about the disease. *	☐	☐	☐	☐	☐

~ Thank you sincerely for your participation ~
