# Supplementary material for: Knowledge, Attitudes, and Practices Regarding Breast Cancer Screening Among Females in Saudi Arabia
Source: Healthcare (Basel). 2026 Jul 6;14(13):2003. doi: 10.3390/healthcare14132003 (PMC13362469; doi:10.3390/healthcare14132003)
Supplement: Supplementary file 1 [file healthcare-14-02003-s001.zip › S2_STROBE Checklist.pdf]

Supplementary File S2

STROBE Checklist for Cross-Sectional Studies

Knowledge, Attitudes, and Practices Regarding Breast Cancer Screening Among Females in Saudi Arabia

Nawaf W. Alruwaili[1,*], Abdullah Mohammed Alfehaid[1,2], Khaled Abdullah Shafi Al-Toum[1,3], Aljazi Bin Zarah[1] and Nora Alafif[1]

[1] Department of Community Health Sciences, College of Applied Medical Sciences, King Saud University, Riyadh 11433, Saudi Arabia; 445911983@student.ksu.edu.sa (A.M.A.); kaltoum@moh.gov.sa (K.A.S.A.-T.); abinzaraah@ksu.edu.sa (A.B.Z.); nalafeef@ksu.edu.sa (N.A.)

[2] Public Health Authority, Riyadh 13352, Saudi Arabia

[3] Ministry of Health, Riyadh 12822, Saudi Arabia

Reference: von Elm E, et al. The STROBE Statement. *PLoS Med.* 2007, 4, e296. https://doi.org/10.1371/journal.pmed.0040296

#	Item	STROBE Requirement	Reported In Manuscript — Status	Location
**TITLE AND ABSTRACT**				
1	Title and abstract	Indicate design; informative summary	Title: 'Cross-Sectional Study' ✓. Abstract: structured ~250 words (Healthcare guideline; current count: 275 words — marginally over, typical for structured peer-review abstracts); Background/Methods/Results/Conclusions headings present as required by Healthcare; age-stratified mammography rate included ✓	Title; Abstract
**INTRODUCTION**				
2	Background/Rationale	Explain scientific background	Introduction §1–4: Saudi Cancer Registry 2020 [20]; 2002–2022 incidence trends [32]; WHO GBCI benchmark [10]; CHBMS framework [13–15]; socioeconomic disparities [32]; evidence gap stated ✓	Introduction §1–4
3	Objectives	State specific objectives	Introduction §5: four numbered objectives including age-stratified uptake ✓	Introduction §5
**METHODS**				
4	Study design	Present key elements	Section 2.1: 'cross-sectional study'; Dec 2024–Feb 2025; STROBE adherence stated ✓	Section 2.1
5	Setting	Setting, locations, dates	All 13 Saudi regions named; KSU institutional email + social media + in-person ✓	Section 2.1
6	Participants	Eligibility, sources, methods	Females ≥20 years residing in Saudi Arabia (Saudi + resident non-Saudi);	Section 2.2

#	Item	STROBE Requirement	Reported In Manuscript — Status	Location
			quota-based design with pre-specified regional and age-stratum targets; facility-based (PHC/hospital/community) + online (KSU email/social media) recruitment; not probability sampling ✓	
7	Variables	Define all key variables	Primary outcome: any screening in preceding 5 years, explicitly coded screened=1/not screened=0; mammography and ≥40 mammography subgroup as labeled secondary outcomes; 8 regression predictors; CHBMS constructs ✓	Sections 2.3, 2.6
8	Data sources/Measurement	Sources and methods	CHBMS [13, 14] + Arabic validation [15]; BCAM [30]; CVI=0.91; KR-20(knowledge)=0.45; α(attitude)=0.74; item-total r: −0.11–0.40; 7-of-19-item subset rationale given ✓	Section 2.3
9	Bias	Address potential bias	Social desirability; non-probability recruitment/selection bias (reordered to lead Limitations); family-history item not skip-logic-gated (denominator corrected, disclosed); 9.4% any-screening vs. mammography internal inconsistency disclosed; response rate (~89% of approached) reported; MCAR analysis (Section 3.6); cautious experiential-reporting reinterpretation of positive barrier ORs, empirically checked against raw-data coding ✓	Sections 2.6, 3.6, 4.3, 4.5
10	Study size	Explain derivation	Cochran's formula: n=384 minimum; 10% inflation to n=426; achieved in full ✓	Section 2.2
11	Quantitative variables	How variables handled	Knowledge dichotomized ≥5; ordinal age/income predictors (dummy-coded sensitivity in Table S6); Cramér's V effect sizes; small-cell categories (age ≥60; income >20,000 SAR) merged for chi-square testing; Shapiro–Wilk confirms non-normal knowledge score (W=0.94, p<0.001) → median/IQR + Mann–Whitney/Kruskal–Wallis used ✓	Section 2.6
12	Statistical methods	All methods; subgroups; missing; sensitivity	Chi-square (merged categories where needed) + Cramér's V; logistic regression (enter, justified); VIF 1.07–2.43; Hosmer–Lemeshow; MCAR test; dummy-coded sensitivity (Table S6); age-restricted mammography-specific sensitivity (Table S7, non-significant, transparently	Section 2.6

#	Item	STROBE Requirement	Reported In Manuscript — Status	Location
			reported); marital-status sensitivity (Table S5) ✓	
**RESULTS**				
13	Participants	Numbers; reasons; flow diagram	N=426 (100% quota). S3 Figure S1 flow diagram ✓	Results; S3 Fig S1
14	Descriptive data	Characteristics; missing data	Table 1: sociodemographics. Table 6 (Section 3.6): MCAR analysis ✓	Tables 1, 6
15	Outcome data	Outcome events	Tables 3, 4 (age-stratified), 5 ✓	Tables 3–5
16	Main results	Estimates; effect sizes	Table 7: χ^2+Cramér's V. Table 8: OR+95%CI+Wald ✓	Tables 7–8
17	Other analyses	Subgroups; sensitivity	Table 4: age-stratified rates (cross-tabulation). Tables S1–S7 + Figure S1 (S3), including new dummy-coded (S6) and mammography-specific age-restricted (S7) sensitivity analyses ✓	Table 4; S3
**DISCUSSION**				
18	Key results	Summarize by objectives	Discussion §4 opening + §4.1–4.4: all objectives addressed ✓	Discussion §4
19	Limitations	Bias; imprecision	Section 4.5, tightened from 368 to 162 words; restructured to lead with non-probability recruitment/selection bias; all limitations retained concisely (cross-sectional design, self-report, skip-logic gap, 9.4% inconsistency, self-efficacy absence, KR-20=0.45); future-research sentence retained; no cross-references within the paragraph ✓	Section 4.5
20	Interpretation	Cautious; compare with evidence	CHBMS framework; ≥15 comparative studies; HBM theoretical framing; causal hedging; response-shift cited [25] ✓	Discussion §4.1–4.4
21	Generalizability	External validity	Reframed: geographic breadth across 13 administrative regions presented as a strength, but explicitly not equated with national representativeness; quota-based recruitment and sampling over-representation (younger, educated, urban) discussed as limiting external validity ✓	Section 4.5
**OTHER**				
22	Funding	Source and role	ORF-2026-1562, KSU; funders had no role ✓	Funding
