# Supplementary material for: Knowledge, Attitudes, and Practices Regarding Breast Cancer Screening Among Females in Saudi Arabia
Source: Healthcare (Basel). 2026 Jul 6;14(13):2003. doi: 10.3390/healthcare14132003 (PMC13362469; doi:10.3390/healthcare14132003)
Supplement: Supplementary file 1 [file healthcare-14-02003-s001.zip › S3_Supplementary Tables.pdf]

Supplementary File S3

Supplementary Statistical Tables and Figures

Knowledge, Attitudes, and Practices Regarding Breast Cancer Screening Among Females in Saudi Arabia

Nawaf W. Alruwaili[1,*], Abdullah Mohammed Alfehaid[1,2], Khaled Abdullah Shafi Al-Toum[1,3], Aljazi Bin Zarah[1] and Nora Alafif[1]

[1] *Department of Community Health Sciences, College of Applied Medical Sciences, King Saud University, Riyadh 11433, Saudi Arabia; 445911983@student.ksu.edu.sa (A.M.A.); kaltoum@moh.gov.sa (K.A.S.A.-T.); abinzaraah@ksu.edu.sa (A.B.Z.); nalafeef@ksu.edu.sa (N.A.)*

[2] *Public Health Authority, Riyadh 13352, Saudi Arabia*

[3] *Ministry of Health, Riyadh 12822, Saudi Arabia*

Table S1. Composite knowledge score by age group (n = 426).

Age Group	n	Mean ± SD	Median	Range	High Knowledge ≥5 (%)	Item-Total r Range
20–29 years	182	4.37 ± 1.57	4	0–7	48.9	−0.11–0.40
30–39 years	108	4.54 ± 1.51	5	1–7	55.6	−0.11–0.40
40–49 years	70	4.57 ± 1.51	5	0–7	55.7	−0.11–0.40
50–59 years	57	4.81 ± 1.30	5	1–7	61.4	−0.11–0.40
≥60 years	9	4.89 ± 1.96	6	0–6	77.8	−0.11–0.40
Total	426	4.51 ± 1.52	5	0–7	54.0	−0.11–0.40

Kruskal–Wallis test (used in place of ANOVA as the knowledge score departs significantly from normality; Shapiro–Wilk W = 0.94, $p < 0.001$; see Section 2.6 and main manuscript): $H(4) = 5.995$, $p = 0.200$. High knowledge ≥5/7; KR-20 = 0.45; item-total r range: −0.11 to 0.40 (five of seven items exceed the 0.20 minimum threshold [25]; see main manuscript Section 2.3.4 for discussion of the heterogeneous, multi-domain item content underlying this reliability estimate).

Table S2. Breast cancer screening uptake (any method, preceding 5 years) by administrative region (n = 426).

Administrative Region	n	Screened—n (%)	Not Screened—n (%)
Riyadh	87	25 (28.7)	62 (71.3)
Qassim	50	10 (20.0)	40 (80.0)
Makkah	36	13 (36.1)	23 (63.9)
Tabuk	36	20 (55.6)	16 (44.4)
Najran	37	22 (59.5)	15 (40.5)
Eastern Province	32	11 (34.4)	21 (65.6)
Madinah	29	13 (44.8)	16 (55.2)
Northern Borders	26	20 (76.9)	6 (23.1)
Ha'il	22	15 (68.2)	7 (31.8)
Asir	20	10 (50.0)	10 (50.0)

Administrative Region	n	Screened—n (%)	Not Screened—n (%)
Al-Jouf	19	11 (57.9)	8 (42.1)
Al-Baha	16	11 (68.8)	5 (31.3)
Jazan	16	6 (37.5)	10 (62.5)
Total	426	187 (43.9)	239 (56.1)

Regional chi-square not computed (small cell sizes). Qassim (20.0%) and Riyadh (28.7%) had the lowest uptake; Northern Borders (76.9%) and Al-Baha (68.8%) the highest.

Table S3. Breast cancer screening uptake by marital status (n = 426).

Marital Status	n	Screened—n (%)	Not Screened—n (%)	χ^2	p-Value
Single	179	39 (21.8)	140 (78.2)		
Married	170	97 (57.1)	73 (42.9)		
Divorced	64	42 (65.6)	22 (34.4)		
Widowed	13	9 (69.2)	4 (30.8)		
Total	426	187 (43.9)	239 (56.1)	63.14 (df=3)	<0.001 ***

$\chi^2(3)=63.14$, $p<0.001$. Differences reflect age confounding: single participants were predominantly aged 20–29. *** $p<0.001$.

Table S4. Item-level frequency distribution and item-total correlations for composite knowledge score (n = 426).

Knowledge Item	Correct	Correct—n (%)	Incorrect—n (%)	Not Sure—n (%)	Corrected r
Aware of breast cancer	Yes	367 (86.2)	59 (13.8)	—	0.25
Breast cancer is treatable	Yes	229 (53.8)	87 (20.4)	110 (25.8)	0.14
Breast cancer is NOT contagious	No	288 (67.6)	100 (23.5)	38 (8.9)	−0.11
Obesity increases risk	Yes	180 (42.3)	113 (26.5)	133 (31.2)	0.31
Physical inactivity increases risk	Yes	223 (52.3)	70 (16.4)	133 (31.2)	0.31
Smoking increases risk	Yes	255 (59.9)	68 (15.9)	103 (24.2)	0.40
Early detection is important	Yes	379 (88.7)	47 (11.3)	—	0.23

Corrected item-total r: five of seven items ≥0.20 (minimum acceptable threshold [25]); the contagion-misconception item shows negative discrimination (−0.11). KR-20 = 0.45 (Kuder–Richardson 20, the dichotomous-item equivalent of Cronbach's α); this lower-than-conventional value reflects the scale's deliberately heterogeneous, multi-domain factual content rather than poor item construction (see main manuscript Section 2.3.4).

Table S5. Sensitivity analysis: binary logistic regression including marital status (n = 426).

Variable	β	OR	95% CI	p-Value	Sig.
Age group	0.351	1.42	1.13–1.79	0.003	**
Monthly income	0.430	1.54	1.22–1.94	<0.001	***
Marital: Married (ref=Single)	0.611	1.84	0.98–3.46	0.058	†
Marital: Divorced (ref=Single)	0.720	2.05	0.99–4.27	0.054	†
Marital: Widowed (ref=Single)	0.703	2.02	0.56–7.34	0.288	ns
High knowledge score	0.291	1.34	0.80–2.24	0.269	ns
Transportation barrier	1.186	3.27	1.87–5.73	<0.001	***
Family discouragement	1.063	2.90	1.63–5.15	<0.001	***

Sensitivity model: $\chi^2(11)=195.20$, $p<0.001$; McFadden's pseudo $R^2=0.332$; Hosmer-Lemeshow p=0.412. Primary predictor estimates unchanged—primary model robustness confirmed. † p<0.10; ns, non-significant; ** p<0.01; *** p<0.001.

Table S6. Sensitivity analysis: binary logistic regression with age and monthly income entered as dummy-coded categorical variables (reference: 20–29 years; ≤5,000 SAR) (n = 426).

Variable	β	OR	95% CI	p-Value	Sig.
Age 30–39 (ref: 20–29)	0.931	2.54	1.30–4.97	0.007	**
Age 40–49 (ref: 20–29)	1.381	3.98	1.82–8.69	<0.001	***
Age 50–59 (ref: 20–29)	1.353	3.87	1.66–8.99	0.002	**
Age ≥60 (ref: 20–29)	-0.062	0.94	0.18–4.91	0.942	ns
Income 5,001–10,000 SAR (ref: ≤5,000)	0.504	1.66	0.83–3.30	0.152	ns

Variable	β	OR	95% CI	p-Value	Sig.
Income 10,001–15,000 SAR (ref: ≤5,000)	1.203	3.33	1.58–7.03	0.002	**
Income 15,001–20,000 SAR (ref: ≤5,000)	1.528	4.61	1.69–12.54	0.003	**
Income >20,000 SAR (ref: ≤5,000)	0.140	1.15	0.22–5.93	0.868	ns
High knowledge score	0.427	1.53	0.91–2.58	0.110	ns
Fear of diagnosis	0.204	1.23	0.59–2.55	0.586	ns
Radiation concern	0.017	1.02	0.51–2.04	0.962	ns
Pain anxiety	0.550	1.73	0.90–3.33	0.099	ns
Transportation barrier	1.182	3.26	1.84–5.76	<0.001	***
Family discouragement	1.080	2.95	1.63–5.31	<0.001	***

β, regression coefficient; OR, adjusted odds ratio; CI, confidence interval. This dummy-coded specification tests the linearity assumption underlying the ordinal coding used in the main manuscript's Table 8. The direction and significance of the transportation-barrier and family-discouragement associations are reproduced; the age ≥60 years and income >20,000 SAR strata (the smallest cells, n = 9 and n = 12 respectively) are non-significant, consistent with limited power rather than a reversal of the broader dose-response pattern seen across the intermediate categories.

Table S7. Sensitivity analysis: binary logistic regression for mammography-specific uptake restricted to age-eligible women (≥40 years) (n = 136). Overall model not statistically significant (likelihood-ratio $\chi^2(8) = 16.51$, $p = 0.208$; pseudo $R^2 = 0.058$).

Variable	β	OR	95% CI	p-Value	Sig.
Age group (ordinal, 3 levels within ≥40)	-0.428	0.65	0.36–1.18	0.155	ns
Monthly income (ordinal)	-0.112	0.89	0.62–1.30	0.557	ns
High knowledge score	-0.307	0.74	0.35–1.56	0.423	ns
Fear of diagnosis	0.266	1.31	0.47–3.64	0.611	ns

Variable	β	OR	95% CI	p-Value	Sig.
Radiation concern	-0.140	0.87	0.31–2.46	0.791	ns
Pain anxiety	-0.648	0.52	0.17–1.60	0.257	ns
Transportation barrier	0.843	2.32	0.95–5.69	0.065	ns
Family discouragement	0.451	1.57	0.70–3.54	0.277	ns

β, regression coefficient; OR, adjusted odds ratio; CI, confidence interval. Unlike the primary model (Table 8, main manuscript), which uses any screening as the outcome in the full sample, this model restricts both the sample (to age-eligible women, n = 136) and the outcome (to mammography specifically). No predictor reached significance at α = 0.05; transportation barrier was closest (OR = 2.32, p = 0.065). This null finding is reported transparently and should be read alongside the full-sample result rather than treated as contradicting it (see main manuscript Sections 3.8 and 4.2).

Supplementary Figure S1. Participant Recruitment and Inclusion Flow Diagram.

Questionnaires distributed (online + in-person, all 13 regions) n = 480

▼

✖ Declined to participate: n = 52 | Incomplete/ineligible responses: n = 2

▼

Completed questionnaires received n = 428

▼

✖ Post-submission withdrawal of consent: n = 2 (participants requested data removal after submission but before data entry; these two withdrawals are distinct from the initial two incomplete/ineligible exclusions)

▼

Final analysis sample N = 426

Figure S1. Participant recruitment, screening, and inclusion flow diagram. All 13 regional quotas achieved. Post-submission withdrawals (n=2) were distinct from initial incomplete/ineligible exclusions (n=2), yielding 426 in the final sample.

Software and Reproducibility Note

All primary analyses: IBM SPSS Statistics Version 26.0 (IBM Corp., Armonk, NY, USA). During revision, key statistics (reliability, Shapiro–Wilk, chi-square, and logistic regression coefficients) were independently cross-checked in Python (pandas/SciPy/statsmodels) against the raw response data and reproduced to 2–3 decimal places. Age-stratified rates (Table 4, main manuscript): cross-tabulation of age group against screening uptake. KR-20 and item-total correlations (Table S4): computed directly from dichotomous item responses (Kuder–Richardson 20 formula). Knowledge-score group comparisons (Table S1) use the Kruskal–Wallis test in place of ANOVA,

reflecting the non-normal distribution of the bounded composite score (Shapiro–Wilk $W = 0.94$, $p < 0.001$). MCAR analyses (Section 3.6, main manuscript): independent-samples t-test and Pearson chi-square. Raw data and analysis scripts available from the corresponding author (nalruwaili@ksu.edu.sa) upon reasonable request.

Key References for Supplementary Files

7. World Health Organization. Global Breast Cancer Initiative Implementation Framework: Assessing, Strengthening and Scaling Up of Services for the Early Detection and Management of Breast Cancer; WHO: Geneva, Switzerland, 2023. Available online: https://www.who.int/publications/i/item/9789240074576 (accessed on 15 March 2025).

8. AlSaleh, K.A. Efficacy of breast cancer screening program in the Kingdom of Saudi Arabia. Saudi Med. J. 2022, 43, 428–432. https://doi.org/10.15537/smj.2022.43.4.20210941

10. Champion, V.L. Instrument refinement for breast cancer screening behaviors. Nurs. Res. 1993, 42, 139–143. https://doi.org/10.1097/00006199-199305000-00004

11. Champion, V.L. Revised susceptibility, benefits, and barriers scale for mammography screening. Res. Nurs. Health 1999, 22, 341–348. https://doi.org/10.1002/(SICI)1098-240X(199908)22:4<341::AID-NUR8>3.0.CO;2-P

12. Mikhail, B.I.; Petro-Nustas, W.I. Transcultural adaptation of Champion's Health Belief Model Scales. J. Nurs. Scholarsh. 2001, 33, 159–165. https://doi.org/10.1111/j.1547-5069.2001.00159.x

21. Linsell, L.; Forbes, L.J.L.; Burgess, C.; Kapari, M.; Thurnham, A.; Ramirez, A.J. Validation of a measurement tool to assess awareness of breast cancer. Eur. J. Cancer 2010, 46, 1374–1381. https://doi.org/10.1016/j.ejca.2010.02.034

37. Al-Hanawi, M.K.; Hashmi, R.; Almubark, S.; Qattan, A.M.N.; Pulok, M.H. Socioeconomic inequalities in uptake of breast cancer screening among Saudi women: A cross-sectional analysis of a national survey. Int. J. Environ. Res. Public Health 2020, 17, 2056. https://doi.org/10.3390/ijerph17062056

38. Saudi Health Council. Cancer Incidence Report Saudi Arabia 2020; National Cancer Center, Saudi Health Council: Riyadh, Saudi Arabia, 2022. Available online: https://shc.gov.sa (accessed on 15 March 2025).

43. Alghofaili, M.; Almutairi, S.; Alotaibi, A.; Alshehri, A.; Alshahrani, A. Sociodemographic, behavioral, and psychosocial factors associated with mammography screening uptake among women in Saudi Arabia: A cross-sectional survey. Front. Public Health 2025, 13, 1584327. Available online: https://www.ncbi.nlm.nih.gov/pmc/articles/PMC12250423/ (accessed on 28 May 2026).

44. National Cancer Institute. Surveillance, Epidemiology, and End Results (SEER) Program. Cancer Stat Facts: Female Breast Cancer. National Cancer Institute: Bethesda, MD, USA, 2024. Available online: https://seer.cancer.gov/statfacts/html/breast.html (accessed on 15 March 2025).

45. Nunnally, J.C.; Bernstein, I.H. Psychometric Theory, 3rd ed.; McGraw-Hill: New York, NY, USA, 1994.
